# Assessing the national burden of allergic asthma by web-search data, pollen counts, and drug prescriptions in Germany and Sweden^☆^

**DOI:** 10.1016/j.waojou.2023.100752

**Published:** 2023-02-23

**Authors:** Sebastian Sitaru, Linda Tizek, Jeroen Buters, Agneta Ekebom, Jan-Erik Wallin, Alexander Zink

**Affiliations:** a Technical University of Munich, School of Medicine, Department of Dermatology and Allergy, Biedersteiner Str. 29, 80802 Munich, Germany; b Center Allergy & Environment (ZAUM), Member of the German Center for Lung Research (DZL), Technical University Munich/Helmholtz Center Munich, Munich, Germany; c Department of Environmental Research and Monitoring, Swedish Museum of Natural History, Stockholm, Sweden; d Pollen Laboratory in Umeå Ltd, Umeå, Sweden; e Division of Dermatology and Venereology, Department of Medicine Solna, Karolinska Institutet, 17176 Stockholm, Sweden

**Keywords:** Allergic asthma, Google, Pollen, Climate, Drug prescriptions

## Abstract

**Background:**

Asthma and its main phenotype allergic asthma are prevalent, chronic, and complex diseases affecting 4% of the population. One main trigger for allergic asthma exacerbations is pollen. Online health information search behavior by people is increasing, and analysis of web-search data can provide valuable insight into disease burden and risk factors of a population.

**Objectives:**

We sought to perform a web-search data analysis and correlation to climate factors and pollen in 2 European countries.

**Methods:**

We analyzed the national web-search volume for allergic asthma-related keywords in Germany and Sweden from 2018 to 2021 and correlated it to local pollen counts, climatic factors, and drug prescription rates.

**Results:**

Per capita, more searches were conducted in Sweden than in Germany. A complex geographic stratification within the countries was observed. Search results were seasonal with a peak in spring and correlated with pollen counts in both countries. However, anti-asthmatic drug prescription rates in Sweden, as well as temperature and precipitation in both countries, did not correlate with search volume.

**Conclusion:**

Our analysis offers population-level insights about this complex disease by reporting its needs and establishing the correlation to pollen counts, which enables a targeted approach in the public health management of allergic asthma. Local pollen counts, as opposed to temperature or precipitation, might be good predictors of allergic asthma disease burden.

## Introduction

With a prevalence of approximately 4%, asthma is among the 5 most common chronic diseases in the world, affecting adults and children alike.^1^ This disease and its most common phenotype, allergic asthma, are complex, heterogeneous conditions, whereby external stimuli together with a genetic predisposition lead to airway inflammation that is characterized by bronchial hyperresponsiveness, obstruction, and airway remodeling, causing wheezing, coughing, and dyspnea amongst other symptoms.^1^^,^^2^ In allergic asthma, patients display sensitizations to environmental allergens and an association between exposure and asthma symptom exacerbation.^1^^,^^2^ This leads to seasonal variations in allergic asthma disease burden with a peak in spring in the northern hemisphere.^3^ Common environmental allergens include pet dander, house dust mites, cockroaches, mold spores, and pollens.^2^

The main goal of asthma therapy is to reduce the number and severity of exacerbations with the least side effects.^1^ Classic treatment intervenes in 2 pathophysiological processes: bronchodilators (especially beta2-adrenergic agonists) for acute attacks and anti-inflammatory drugs (especially inhaled corticosteroids) for middle-to-long-term prevention of attacks. For allergic asthma, specific allergen immunotherapy also improves disease severity and course.^4^^,^^5^

Due to its prevalence and complexity, allergic asthma is a major burden for health care systems and affected individuals.^6^^,^^7^ For example, asthma including allergic asthma, is both an underdiagnosed and overdiagnosed disease in many parts of the world, which can lead to poorer quality of life, more hospitalizations, but also more side-effects and higher drug costs, amongst others.^8^, ^9^, ^10^, ^11^

A valuable resource to gain insights into the needs and interests of a population regarding a disease is the analysis of Google web-search data.^12^, ^13^, ^14^, ^15^, ^16^, ^17^ In addition to consulting doctors, more and more people are using the internet to search for health-related information.^18^ For this study, data from Sweden and Germany were used. Germany and Sweden are aligned along a north-south axis, which allows for better comparisons of climatic influencing factors. Furthermore, they have similar rates of online health information search behavior: In Sweden, nearly 70% of the population and in Germany nearly 57% have used the internet for health-related information.^19^ As a portal to web information, the main search engine in both countries is Google by a large margin.^20^^,^^21^ In Germany in a cross-sectional study from 2009 to 2012, 15.6% of children had at least 1 atopic disease^22^ of which 4.1% had asthma, while in Sweden in a similar study, 32% of participants had at least 1 allergy and 9.8% asthma.^23^ At the time of writing, Germany has a population of around 83 million inhabitants and Sweden has 1 of approximately 10 million.^24^

Asthma exacerbations can be correlated to climate parameters and pollen counts. Asthma, including allergic asthma disease burden negatively correlated to mean temperature.^25^^,^^26^ Another study found a complex U-shaped association between asthma disease burden and temperature.^27^ Small real-world studies demonstrated that increased pollen exposure was associated with an increased prevalence of asthma exacerbations.^28^

The aim of this study therefore was to analyze Google web-search data of allergic asthma-related keywords in Germany and Sweden to gain insight into health information seeking behavior of the population and to correlate these data to environmental and other factors, like pollen counts and temperature, to improve the understanding of allergic asthma in terms of risk factors, time course, unmet needs, and geographic hotspots.

## Methods

### Data sources

Google Ads Keyword Planner was used to assess the search volume regarding “allergic asthma” in Sweden and Germany from January 2018 to December 2021. Google Ads Keyword Planner is a tool originally designed for planning web advertisement campaigns by selecting relevant keywords for a search term, but it can be used to extract search volumes for these keywords as well. The respective German and Swedish lay words for allergic asthma (German: “allergisches Asthma”, Swedish: “Allergisk astma”) were entered into the tool, which provided a list of relevant keywords and their monthly search volume for the last 48 months. Only searches which originated from German or Swedish IPs were included in the analysis.

All identified keywords were qualitatively assessed and categorized into the following categories: 1) General (only general terms), 2) Time (includes points in time), 3) Symptoms (includes terms relating to symptoms), 4) Therapy (includes terms relating to asthma therapy), 5) Medical (includes medical terms other than symptoms or therapy), 6) COVID (includes medical terms related to SARS-CoV-2/COVID-19), 7) Animals (includes other terms), 8) Social (includes terms related to self-help groups and testimonials), and 9) Other (includes other terms not mentioned above). Each search term was classified into exactly 1 category; none were excluded from analysis. The categories were chosen in such a way that a minimum of 2 terms were required to open a category. Other terms that could not be grouped were included in the "other" category.

Climate data for Sweden and its counties were obtained from the Swedish Meteorological and Hydrological Institute. For mean monthly temperature and precipitation, data from 1991 to 2020 were used. For Germany, data from the Climate Data Center of the German weather service were used. For mean monthly temperature, data from 1980 to 2010 were used. For mean monthly precipitation, data from 1920 to 2022 were used.

Additionally for Sweden, data on prescriptions of beta-2-sympathomimetics, inhalative glucocorticoids, and “other systemic drugs for obstructive pulmonary disease” were obtained from the statistics database of the National Board of Health and Welfare (Socialstyrelsen^29^). These data include only information about prescribed drugs filled at pharmacies and not over-the-counter (OTC) drugs. Pollen counts for Sweden were obtained from the Swedish Museum of Natural History, which coordinates pollen research in Sweden. Pollen counts for Sweden were obtained from the Swedish Museum of Natural History, which coordinates pollen sampling in Sweden using Hirst-type 7-days volumetric trap.^30^ Pollen was identified with optical microscopy, using vertical traverses (in Sweden).^31^ Daily average pollen counts are expressed as pollen grains per cubic meter of air (pollen grains/m^3^). Data were only available for the counties of Stockholm, Västerbotten, Södermanland, Jönköping, Gotland, Gävleborg, and Jämtland. For the southern German federal state of Bavaria, pollen data were provided by the Center Allergy and Environment Munich (ZAUM) of the Helmholtz Center Munich. Pollen counts for Bavaria were measured by a network of 8 fully automated, image-recognition based devices. The devices (BAA 500, Helmut Hund GmbH, Wetzlar, Germany) sample air 360° at 1000 L/min, of which 100 L/min is used to trap pollen using a virtual cascade impactor and a sticky surface. The different pollen species were photographed using a CCD camera and automatically classified using an artificial intelligence algorithm as is described in detail in Oteros et al (2020) and Oteros et al (2021).^32^^,^^33^ These obtained pollen counts included common and rarer allergenic pollen species in northern and central Europe, namely Alnus, Artemisia, Betula, Corylus, Plantago, Pocaceae, Rumex, Quercus, and Fraxinus (among others).^34^ For analysis, Bavarian pollen counts from the devices were aggregated by arithmetic mean. For both Bavaria and Sweden, pollen counts were aggregated to Total and Birch pollen.

### Statistical analysis

To compare the search volume of German federal states and of Swedish counties, the search volume was normalized to number per 100 000 inhabitants. For Germany, population data were obtained from the Ministry of Statistics (Statistisches Bundesamt^35^) and for Sweden from Statistics Sweden (Statistiska Centralbyrån^36^).

Statistical analyses were performed using R v4.1.2. Unless otherwise specified, Spearman's *r* via the *rcorr* function was used as a correlation method. For geographical comparisons, the Kruskal-Wallis rank sum test was used. Furthermore, *p* values < 0.05 were considered statistically significant (∗) and *p* values < 0.01 were considered highly statistically significant (∗∗). All other *p* values were regarded as not statistically significant (n.s.).

### Data protection

Since only publicly available data were used, institutional review board approval or informed consent was not applicable.

## Results

### Dataset description

**Fig. 1:**
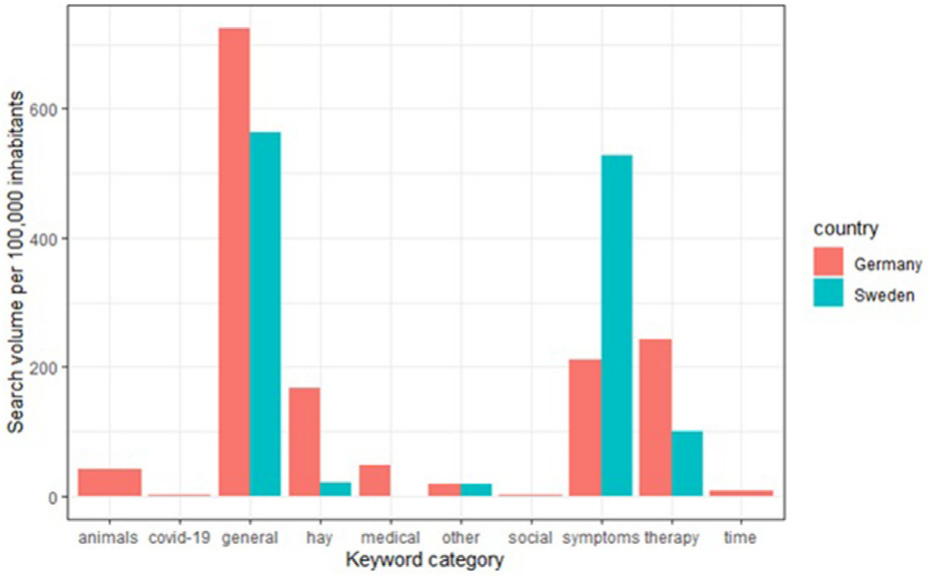
Categorization of the identified keywords. Identified allergic asthma-related keywords were grouped into exactly 1 category, which is shown on the x axis. On the y axis, the number of searches corresponding to the keyword category for Germany (red) and Sweden (blue-green) is shown.

**Fig. 2:**
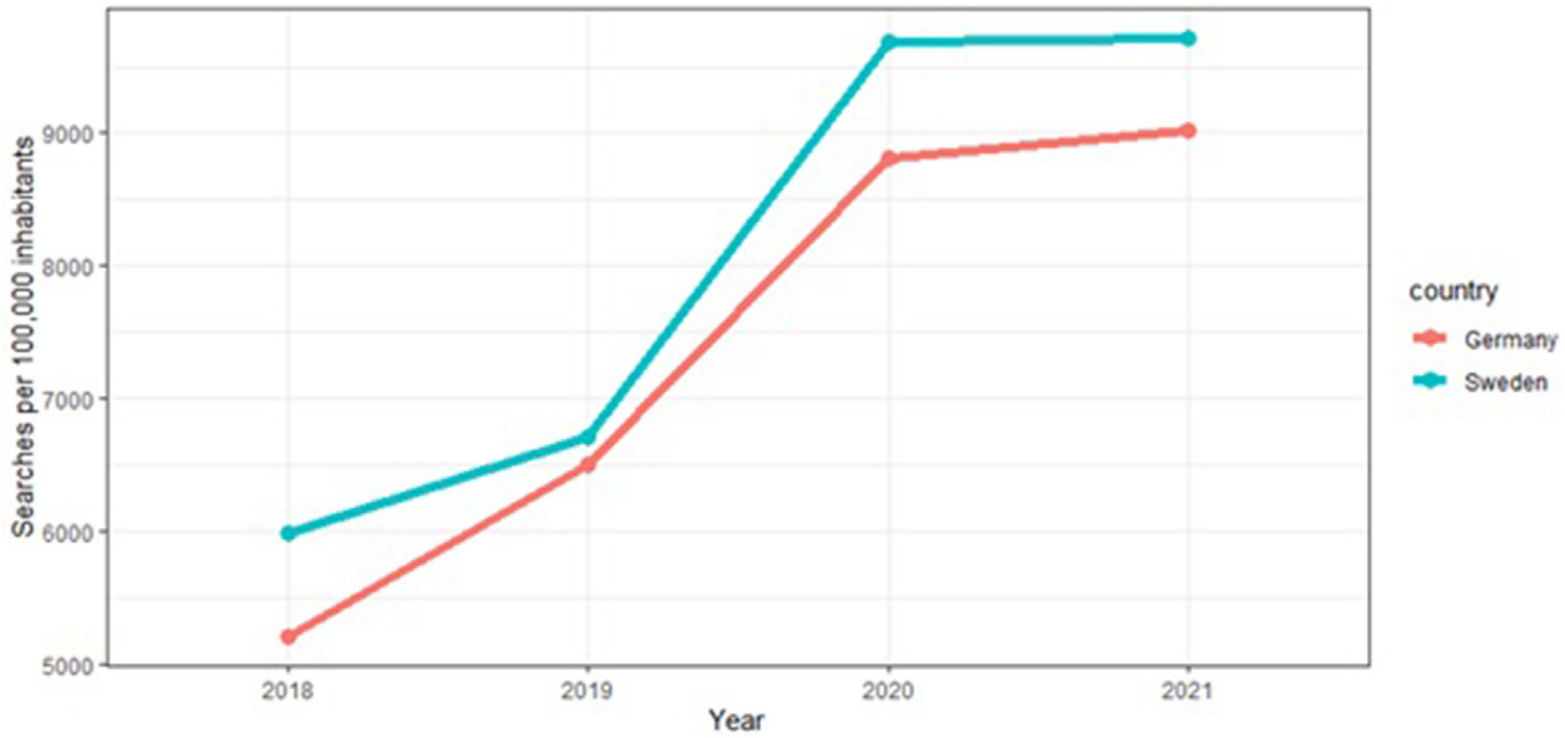
Allergic asthma-related search volume per year in Germany and Sweden. This figure illustrates the total number of searches per 100,000 inhabitants from 2018 to 2021 for Germany (red) and Sweden (blue-green).

For Germany and Sweden, 321 keywords and 24 keywords related to allergic asthma were identified, respectively. In both countries, the highest search volume was observed for the category “general”, followed by the categories “symptom” and “therapy”. In Sweden, the absolute number of symptom-related searches was higher than in Germany (500 vs 200 searches per 100 000 inhabitants). No keywords of time, social, medical, COVID-19, and animal categories were present among the Swedish keywords (Fig. 1). In total, 1,341,800 searches (Germany: 1 214 990, Sweden: 126 810) were recorded for the study period. The mean number of searches per month per 100 000 inhabitants was 642.2 (Germany: 615.7, Sweden: 668.6). During the study period, the search volume initially increased rapidly from 6000 to over 9500 (2018–2020, Sweden) and from 5100 to 8900 (2018–2020, Germany) before levelling off from 2020 to 2021 (Fig. 2).

### Seasonal patterns

**Fig. 3:**
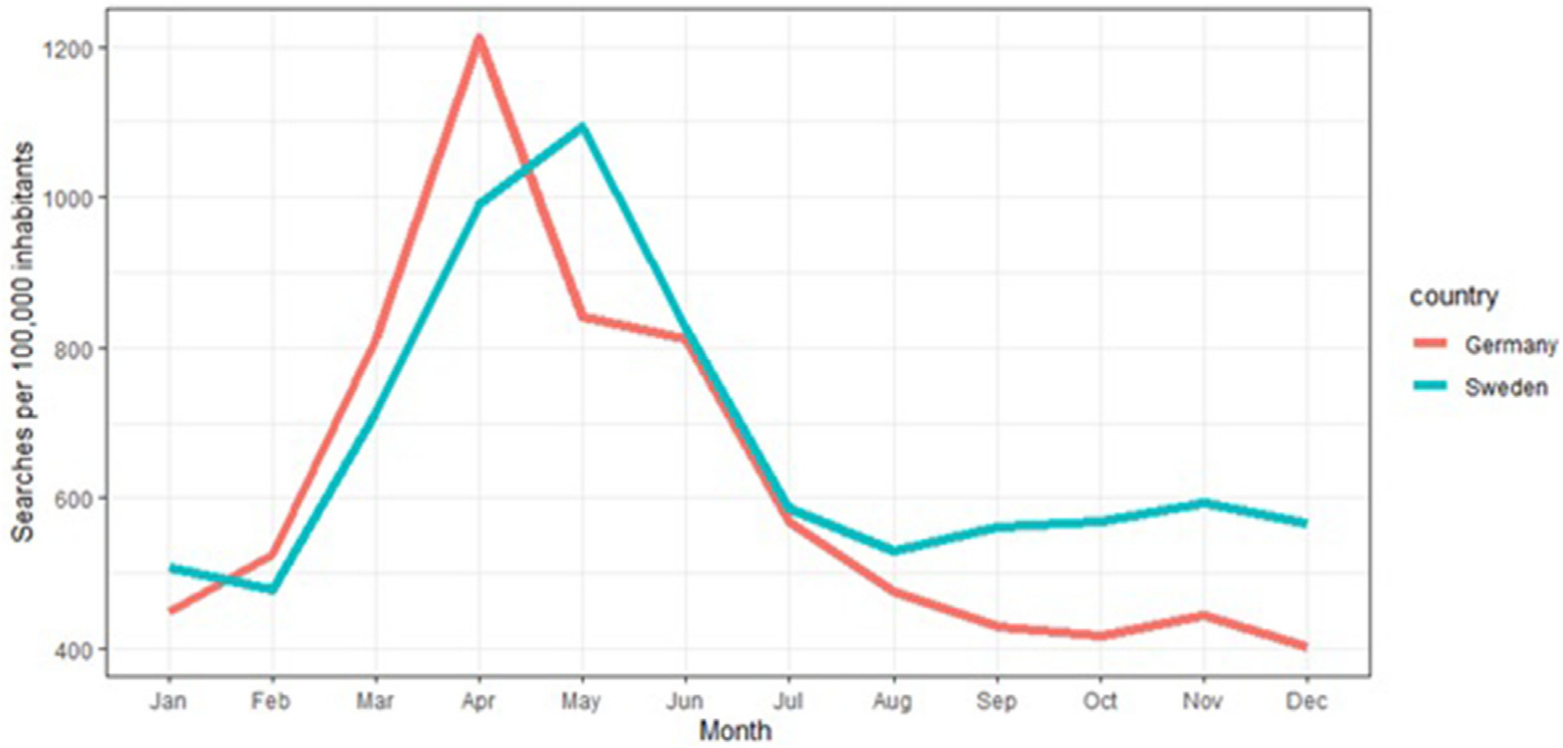
Seasonal variability of allergic asthma-related searches in Germany and Sweden. This line chart shows the average search volume per 100,000 inhabitants in Germany (red) and Sweden (blue-green) in each month. Data was averaged over four years (2018–2021).

Sweden had a higher cumulative search volume per 100 000 inhabitants than Germany over the analyzed period (32 096 searches in Sweden vs 29 553 in Germany). While the average search volume per 100 000 inhabitants was higher in Germany at the beginning of the year and peaked in April (Germany: 1200 searches per 100 000 inhabitants), the search volume was higher in Sweden during the remaining months. In Sweden, a peak in search volume was observed in May (Fig. 3). There was a statistically significant difference of the search volumes in the European spring season from May–March, compared to the rest of the months (p < 0.05 by Wilcoxon ranked sum test, for both German and Swedish datasets).

### Geographical stratification

**Fig. 4:**
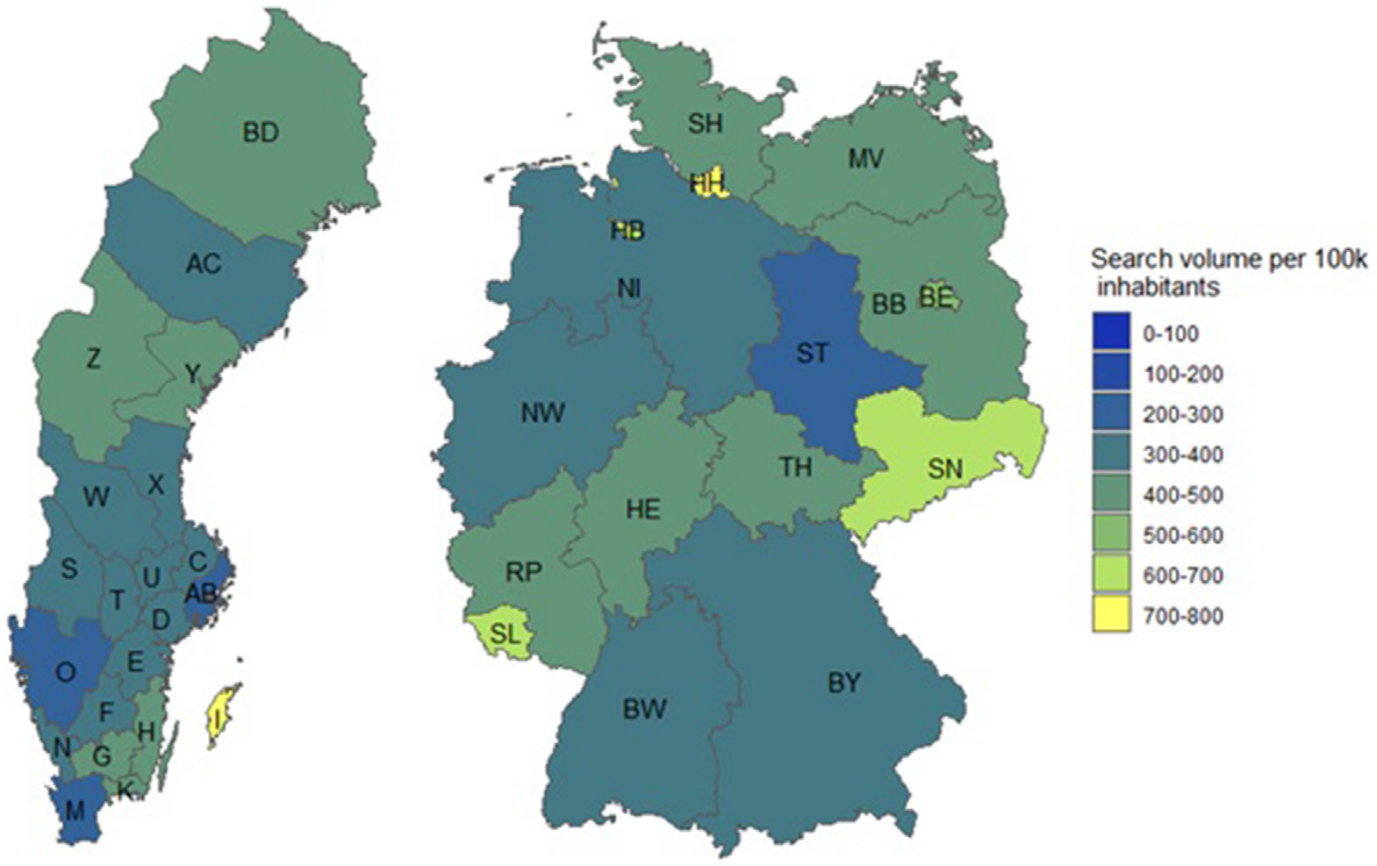
Geographic distribution of search volume within Germany and Sweden. These maps show the geographic distribution of the mean yearly search volume per 100,000 inhabitants in the German (left) and Sweden (right) from 2018 to 2021. The abbreviations correspond to the ISO 3166-2 code of the areas. For Sweden: BD: Norrbottens län, AC: Västerbottens län, Z: Jämtlands län, W: Dalarnas län, S: Värmlands län, O: Västra Götalands län, Y: Västernorrlands län, X: Gävleborgs län, C: Uppsala län, AB: Stockholms län, D: Södermanlands län, E: Östergötlands län, H: Kalmar län, K: Blekinge län, M: Skåne län, N: Hallands län, I: Gotlands län, U: Västmanlands län, F: Jönköpings län, T: Örebro län, G: Kronobergs län. For Germany: SN: Sachsen, BY: Bayern, RP: Rheinland-Pfalz, SL: Saarland, SH: Schleswig-Holstein, NI: Niedersachsen, NW: Nordrhein-Westfalen, BW: Baden-Württemberg, BB: Brandenburg, MV: Mecklenburg-Vorpommern, HB: Bremen, HH: Hamburg, HE: Hessen, TH: Thüringen, ST: Sachsen-Anhalt, BE: Berlin.

Fig. 4 shows the geographic distribution of the overall search volume within the 2 countries. In Sweden, the number of searches per 100 000 inhabitants was higher in Gotland, Kalmar, and Kronoberg in comparison to that in Skane, Västra Götaland, and Stockholm. In Germany, the highest search volume was observed in Hamburg, while the lowest was observed in Saxony-Anhalt. These differences within the Swedish counties, as well as within the German federal states, as calculated by the Kruskal-Wallis rank sum test, are highly statistically significant (p < 0.01).

## Pollen count

**Fig. 5:**
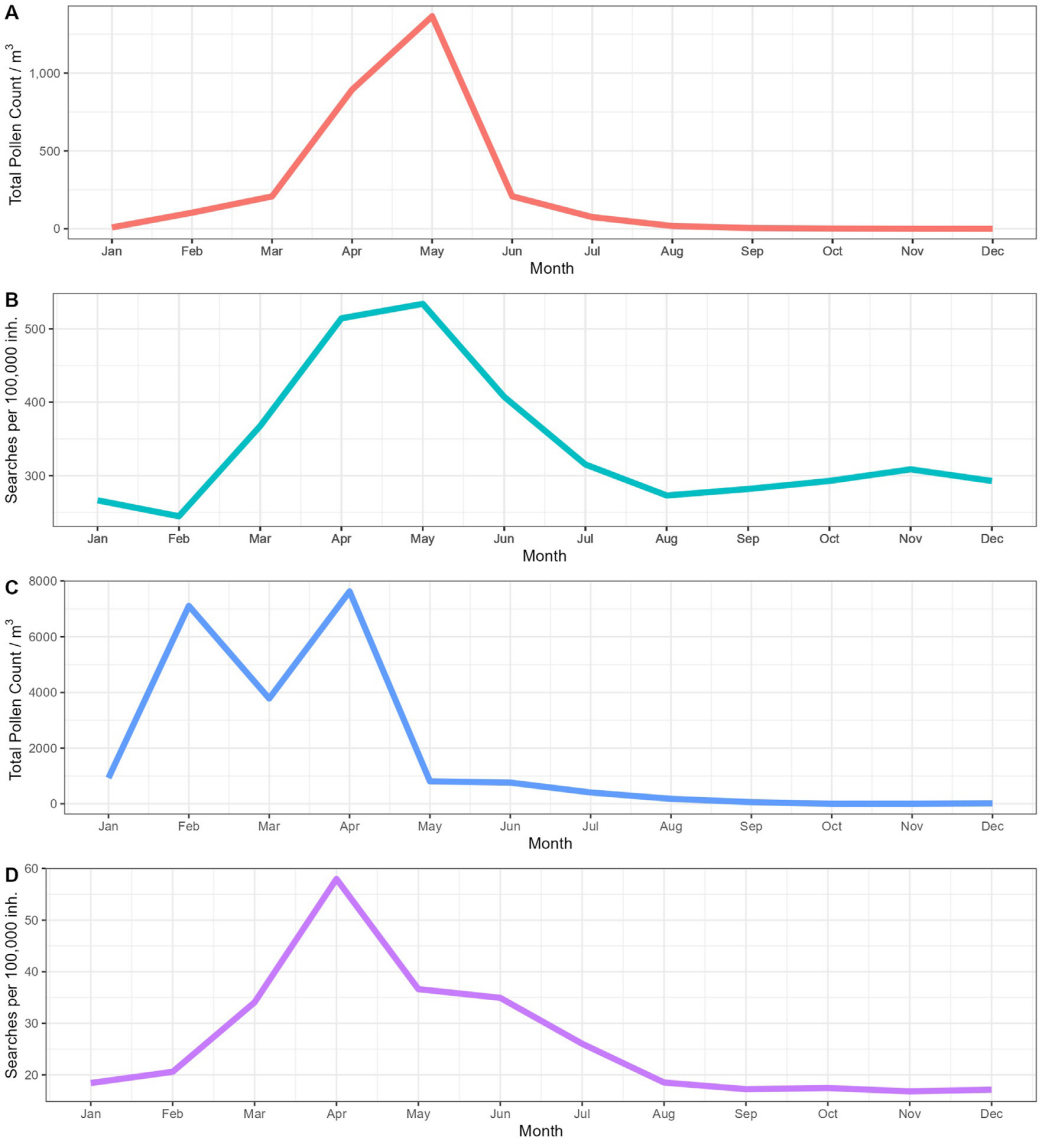
Comparison between pollen counts and search volume in Sweden (A, B) and Bavaria (C, D). Panels A and C show total pollen counts per m^3^, while B and D show search volume per 100,000 inhabitants. Data were averaged over four years (2018–2021). For pollen count, data from 2017 to 2020 were used. For statistical correlation data, see the results section and Table 1.

Fig. 5 shows the pollen counts and search volume in Sweden and in Bavaria, Southern Germany. The curves for Sweden (Fig. 5a, b) positively correlate with the search volume (r = 0.59, p < 0.05), with both showing a peak in May. In Bavaria (Fig. 5c, d), the data correlate strongly (r = 0.76, p < 0.01). The peak in search volume coincides with the peak in pollen count in April. As displayed in Table 1, total pollen counts showed a positive correlation with the search volume in all examined regions. For Jönköping, Södermanland, and Stockholm counties, a correlation coefficient *r* of >0.9 indicated a very strong correlation. When only birch pollen count was analyzed, however, the correlation in Västerbotten, Södermanland, and Jönköping was not statistically significant.

**Table 1:** Correlation of search volume and pollen counts

Region	Pollen type	R	p	
DE-Bavaria	Total	0.76	0.003950	∗∗
SE-Stockholm	Total	0.93	0.000012	∗∗
SE-Västerbotten	Total	0.75	0.004960	∗∗
SE-Södermanland	Total	0.90	0.000066	∗∗
SE-Jönköping	Total	0.91	0.000047	∗∗
SE-Gotland	Total	0.75	0.004926	∗∗
SE-Gävleborg	Total	0.87	0.000255	∗∗
SE-Jämtland	Total	0.62	0.032375	∗
DE-Bavaria	Birch	0.72	0.008825	∗∗
SE-Stockholm	Birch	0.63	0.000063	∗∗
SE-Västerbotten	Birch	0.44	0.317372	n.s.
SE-Södermanland	Birch	0.63	0.096493	n.s.
SE-Jönköping	Birch	0.63	0.051847	n.s.
SE-Gotland	Birch	0.71	0.033579	∗
SE-Gävleborg	Birch	0.86	0.001440	∗∗
SE-Jämtland	Birch	0.94	0.005089	∗∗

*r* = Spearman's *r* correlation coefficient. ∗∗ means highly significant, *p* < 0.01. ∗ means significant, *p* < 0.05. *n.s.* means not significant.

### Correlation with other climate parameters

No significant correlation between mean monthly search volume and mean monthly temperature was observed in Germany (r = 0.2, p = 0.54) and Sweden (r = 0.37, p = 0.24). Similarly, no significant correlation between mean monthly search volume and mean monthly precipitation could be observed in Germany (r = −0.04, p = 0.9) and Sweden (r = −0.16, p = 0.62).

### Correlation with anti-asthmatic drug prescriptions in Sweden

**Fig. 6:**
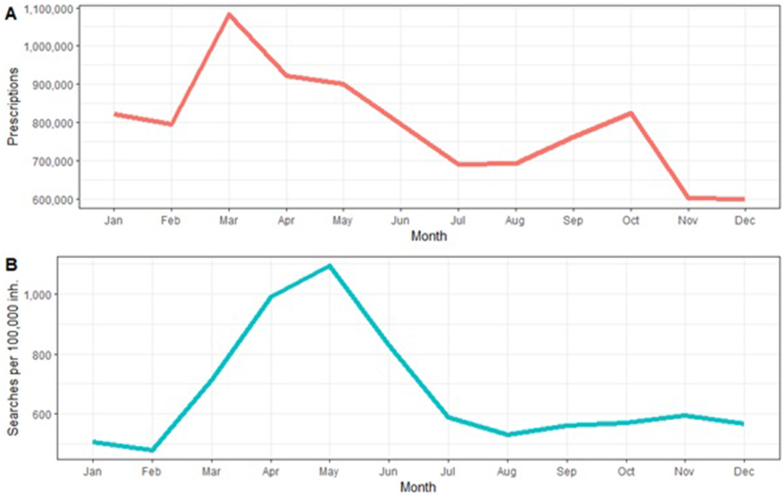
Comparison of anti-asthmatic drug prescriptions (A) and search volume (B) in Sweden. Data is shown per month and was averaged over four years (2018–2021). Relevant drugs included beta-2-sympathomimetics, inhalative glucocorticoids, and “other systemic drugs for obstructive pulmonary disease”.

Upon analyzing the search volume and anti-asthmatic drug prescription rates in Sweden (Fig. 6), no significant correlation was found (r = 0.37, p = 0.24). Drug prescription rates showed 2 peaks, with 1 in March (around 1.1 million prescriptions) and 1 in October (around 850 000 prescriptions). The first drug prescription peak preceded the peak in search volume by 2 months. Similarly, no correlation could be established between the sub-groups of drugs and search volume (Table 2).

**Table 2:** Sub-group analysis of correlation of search volume and drug prescriptions in Sweden

Drug class	r	p	
Glucocorticosteroids (for respiratory tract inflammation)	0.34	0.2755675	n.s.
Selective beta-2-stimulants (bronchodilators)	0.46	0.1309481	n.s.
Other systemic drugs for obstructive pulmonary disease	0.54	0.0666119	n.s.

*r* = Spearman's *r* correlation coefficient. ∗∗ means highly significant, *p* < 0.01. ∗ means significant, *p* < 0.05. *n.s.* means not significant.

## Discussion

The aim of the study was to analyze web-search data of allergic asthma related searches in Germany and Sweden and to correlate them with different climatic and other factors to improve the understanding of allergic asthma. We found more searches per inhabitants in Sweden than in Germany. A statistically significant correlation of search volume with pollen count but not with precipitation or temperature in both countries was found. No correlation was found with drug prescription rates in Sweden.

Compared to searches for other diseases, allergic asthma had similar numbers of average monthly searches as itch, with 717 searches per 100 000 inhabitants.^15^ This suggests that there is an unmet need for information in the general public regarding allergic asthma. Making more high-quality, public information on allergic asthma available may help to empower patients and reduce health care costs.

The mean number of searches per inhabitants per months was higher in Sweden than in Germany. This may be because allergies are more prevalent in Sweden (32% of Swedish children with at least 1 allergy vs 15.6% in Germany) or because Swedish people tend to search more common for information online. For example, 70% of people have used the Internet for healthcare information in Sweden in comparison to 57% in Germany.^19^

Searches about allergic asthma mostly included terms for general information, symptoms, and therapy in both countries. In Sweden, more people searched for symptom-related information than in Germany, while in Germany more people searched for therapy-related information. This may reflect the need for information in the general population and underlines a need for more high-quality information about the disease with a focus on symptoms in Sweden and therapy in Germany. There was a lower number of keywords identified in Swedish searches than in German searches (321 versus 24). The interpretation of this finding is difficult, since the algorithm which generates the keyword lists is not publicly known. However, one possible explanation could be that Swedish search queries were overall less differentiated and more about general information on allergic asthma.

During the study period, search volume increased from 2018 to 2020 before levelling off from 2020 to 2021. One possible explanation for this break in trend could be the COVID-19 pandemic. It was shown that outpatient visits, including for asthma, quickly shifted to the online realm.^37^^,^^38^ It is conceivable that overall, patients resorted to using more online health resources during the pandemic, which also increased search volume. By the end of the year, the ratio of virtual, telemedical visits to total visits had leveled off.^37^^,^^38^ This could, in turn, explain the levelling-off in the search volume we observed.

In both countries, allergic asthma search behavior displayed a seasonal pattern with peaks in spring, while being low during the other seasons. The peak in search volume is higher in Germany. Because the maximum pollen count is also higher in Germany, higher interest in hay-fever related information and possibly increased disease burden may be explaining this higher peak. However, this might not be the only explanation, since it has been demonstrated that pollen can release different amounts of allergens depending on climate and geographic factors, and therefore total pollen count is only 1 variable for total allergen exposure, which ultimately drives allergic diseases.^39^^,^^40^ Nevertheless, a strong overall correlation between pollen counts and search behavior was seen in our study. This is in line with real-world data that linked asthma exacerbations with pollen counts.^28^ Another study identified spring and winter as seasons with a high number of asthma-related emergency department visits.^41^ The winter peak might have been missed by our study since we analyzed the allergic variant of asthma, and exacerbations in winter are usually attributed to the common cold.^42^ The peak in search volume in Sweden occurs about 1 month later than in Germany, which coincides with the peak in pollen count also occurring about 1 month later. Corresponding to findings from previous studies, these data strongly suggest that disease burden in allergic asthma is seasonal, with pollen count being one of the major influencing factors.^28^ Google web-search data can therefore act as a predictor of asthma exacerbations based on local pollen counts.

The most common pollen in central and northern Europe is birch pollen.^43^ In our study, a stronger association was seen with total pollen counts than with birch pollen alone. This finding seems to contradict real-world data, which showed that tree pollen had a much higher chance than grass pollen for causing asthma exacerbations, though this mentioned study did not provide a breakdown of pollen types according species.^28^ Furthermore, the aforementioned study was conducted only in the United States, which may not consider geographic differences of pollen composition or dynamics.

In our study, we could not demonstrate a link between search volume and temperature or precipitation, although earlier studies have shown a link between asthma exacerbations and temperature.^25^^,^^26^ However, these studies pooled data for asthma in general and did not focus on allergic asthma. It is therefore possible that the temperature association only applies for non-allergic asthma. Nevertheless, these findings require further validation with real-world data, such as emergency department visits.

Surprisingly, anti-asthmatic drug prescription rates do not correlate with search behavior in Sweden, with no correlation between drug sub-groups and search volume being observed either. This is not expected, as beta-2-adrenergic drugs are usually used as short-term relievers for asthma. Indeed, real-world data from Sweden showed that medication use was higher during pollen season.^44^ However, it could be theorized that medication use and prescription – which we analyzed – may not be linked and eg, that when the disease burden increases in spring, more of these drugs are normally needed and are being therefore prescribed proactively. Overall, there appears to be a complex relationship between search behavior and drug prescriptions rates. The second peak of drug prescriptions in winter may be explained by the common cold season in winter, which is a known risk factor for asthma exacerbations.^1^

### Limitations

Our study provides insight into national allergic asthma web-search behavior in Sweden and Germany. However, the data only represent web-searches and therefore only represent the intent to obtain information. Due to the nature of search engine web-search data, no information on who conducted the searches is available. Mostly the younger demographic groups use the Internet for health-related information, which surely represents a bias in this study.^19^ Our data are only representative for Sweden and Germany, which are 2 highly developed European countries.

Another limitation of the study is that we were not able to obtain pollen counts for all Swedish counties and for German federal states other than Bavaria. Bavarian pollen data were used as a proxy for German pollen counts. Furthermore, anti-asthmatic drug prescription rates could not be obtained in Germany.

### Summary

In summary, we found disease burden in allergic asthma to be seasonal in both Germany and Sweden, with pollen count being one of the major trigger factors. A direct correlation of disease burden with temperature or precipitation could not be identified. While search volume in both countries followed similar patterns, the search volume per capita was higher in Sweden than in Germany, likely due to higher internet usage for health information or higher disease burden. There was a break in the trend of increasing search volume from 2020 to 2021, which could be related to the start of the COVID-19 pandemic in 2020 and the associated shift in health information seeking behavior. There is a statistically significant geographic stratification within both countries. Interestingly, anti-asthmatic drug prescriptions rates in Sweden did not correlate with search volume, suggesting a more complex interaction between disease burden and drug prescription rates.

## Conclusion and outlook

Overall, our study provides insight into allergic asthma disease burden over time and geographic regions using affordable, readily available web-search data and by identifying influencing variables. It highlights the need for high-quality public information on this disease and could serve as the basis for more targeted campaigns, improving clinical and socioeconomic outcomes of this disease and ultimately the lives of allergic asthma patients.

## Abbreviations

COVID-19, coronavirus disease 2019; OTC, over the counter

## Authors' consent for publication

All authors consent to publication of this manuscript to World Allergy Organization Journal.

## Funding

Competing financial or non-financial interests: Linda Tizek is a current employee of ViiV Healthcare, partly owned by Pfizer and Glaxo-Smith-Kline. Her work for this manuscript was completed before her employment.

### Data availability statement

The data used in this publication can be obtained from the authors upon request.

## Author contributions

SS – Conceptualization, Methodology, Investigation, Data Curation, Writing – original draft, Writing – review & editing, Visualization. LT – Methodology, Formal analysis, Writing – review & editing. JB – Methodology, Data curation, Resources, Writing – review & editing. AE – Resources, data curation, Writing -- review & editing. JEW – Resources, data curation, Writing – review & editing. AZ – Conceptualization, Resources, Writing – review & editing, Supervision.

## Ethics committee approval

Not needed since only publicly available data was used.
